# The Association between Nutritional Markers and Biochemical Parameters and Residual Renal Function in Peritoneal Dialysis Patients

**DOI:** 10.1371/journal.pone.0156423

**Published:** 2016-06-03

**Authors:** Li Li, Wangqun Liang, Ting Ye, Zhenyan Chen, Xuezhi Zuo, Xiang Du, Kun Qian, Chunxiu Zhang, Xiangrong Hu, Junhua Li, Le Wang, Zufu Ma, Ying Yao

**Affiliations:** 1 Department of Clinical Nutrition, Tongji Hospital, Tongji Medical College, Huazhong University of Science and Technology, Wuhan, China; 2 Department of Nephrology, Tongji Hospital, Tongji Medical College, Huazhong University of Science and Technology, Wuhan, China; Emory University, UNITED STATES

## Abstract

Residual renal function (RRF) is an important prognostic factor for peritoneal dialysis patients as it influences the quality of life and mortality. This study was conducted to explore the potential factors correlated with RRF. A cross-sectional study was conducted by recruiting 155 patients with residual GFR more than 1mL/min per 1.73m^2^ at the initiation of peritoneal dialysis. We collected the demographic characteristics, nutritional markers and biochemical parameters of all participants, and analyzed the correlation between these variables and residual GFR as well. The odds ratio of RRF loss associated with each of the nutritional markers and biochemical parameters were estimated by logistic regression model. The residual GFR was negatively correlated with serum phosphate (OR_Q3_ = 2.67, 95%CI: 1.03–6.92; OR_Q4_ = 3.45, 95%CI: 1.35–9.04), magnesium (OR_Q4_ = 3.77, 95%CI: 1.48–3.63), and creatinine (OR_Q3_ = 2.93, 95%CI: 1.09–7.88; OR_Q4_ = 8.64 95%CI: 2.79–26.78), while positively associated with normalized protein catabolic rate (OR_Q3_ = 0.24, 95%CI: 0.09–0.65; OR_Q4_ = 0.11, 95%CI: 0.03–0.35), 24 hours urine volume(OR_Q1_ = 22.87, 95%CI: 2.76–189.24; OR_Q3_ = 0.08, 95%CI: 0.02–0.28) and serum chlorine concentrations (OR_Q1_ = 5.34, 95%CI: 1.94–14.68; OR_Q4_ = 0.28, 95%CI: 0.09–0.85), respectively. Our study suggested that the nutritional markers and biochemical parameters, though not all, but at least in part were closely correlated with RRF in peritoneal dialysis patients.

## Introduction

In recent years, chronic kidney disease (CKD) has become a worldwide public health problem as its rapid increase in the incidence and prevalence. CKD is highly prevalent among adults in both developed [1–3] and developing countries [4]. In 2012, large-scale national survey from China found that the prevalence of CKD was 10.8% [4]. And it was found that 2% of patients with CKD would enter the stage of end-stage renal disease (ESRD), when dialysis or renal transplantation was needed to sustain life. However, most patients prefer to choose dialysis considering the complications after transplantation and side effects of long-term immunosuppressive agents use. Preserving residual renal function (RRF) is important for survival in patients undergoing peritoneal dialysis (PD) or hemodialysis. It has been demonstrated that 1% elevation of the glomerular filtration rate (GFR) decreased mortality 7–48% [5–8]. For Chinese PD patients, a much closer association was found with a higher residual GFR (1 mL/min per 1.73m^2^) reducing 52% relative risk of death [9]. The potential mechanisms underlying these decrease included better fluid removal and blood pressure control, enhanced clearance of middle to large molecular weight uremic toxins, prevention of dialysis-associated amyloidosis caused by the tissue deposition of β2-microglobulin and promotion of hormone synthesis [10–12]. Overhydration with resulting therapy-resistant hypertension and left ventricular hypertrophy is a frequent problem in PD patients [13]. RRF has been proved to be an important determinant in the maintenance of a normal volume status in PD patients [14]. In addition, lower RRF has also been considered as risk factor for depression and impaired health-related quality of life in dialysis patients [15]. Therefore, preservation of RRF is an important goal in the treatment of continuous ambulatory peritoneal dialysis (CAPD) patients.

It has been reported that the loss of RRF was partly due to increased generation of inflammation factors such as C-reactive protein (CRP), intraperitoneal interleukin-6 (IL-6), hyaluronan and neopterin, elevation of the advanced glycation end-product N- "-carboxymethyllysine and fibroblast growth factor 23 (FGF23), and the use of coronary angiography [16–19]. Recent study from Chang *et al* has also found that low serum bicarbonate predicts RRF loss in peritoneal dialysis patients [20].

As an important marker to measure RRF of dialysis patients, glomerular filtration rate (GFR) of residual renal is well correlated with measured total creatinine clearance, less expensive and time-consuming compared to weekly creatinine clearance (wCcr) and Kt/V of residual renal [21]. Thus, the present study was conducted to explore the potential influence factors of residual GFR in Chinese patients with CAPD.

## Materials and Methods

### Ethics

The study has been approved by the Institutional Review Board of Tongji Hosipital, Tongji Medical College, Huazhong University of Science and Technology. All the patients signed the informed consent about the use of their tissue samples and baseline data in research.

### Patients and data collection

This cross-sectional study included 155 end-stage renal disease (ESRD) patients on CAPD treated at Tongji hospital of Wuhan in China from January, 2005 to December, 2015. We retrospectively reviewed their medical records. The inclusion criteria were: 1) having residual GFR more than 1 mL/min per 1.73m^2^ before peritoneal dialysis; 2) being under CAPD treatment as the initial renal replacement treatment for at least 3 years; 3) absence of hypoalbuminemic state (nephritic range proteinuria, advanced liver disease, or intestinal malabsorption); 4) absence of a history of atherosclerotic vascular disease at the initiation of PD. A semi-structured questionnaire was used to collect information about age, gender, occupation, education, marital status, history of hypertension or diabetes, etiology of CKD, duration on PD, type of PD solution, number of exchanges per day and erythropoietin dose, dialysis tubing, height and dry weight. Body weight was preferably done with empty abdominal cavity. The Body Surface Area (BSA) was calculated basing on the formula of Gehan and George [22]. The occupation was classified according to the intensity of laboring. Farmers and workers were categorized as high strength work, office clerks and students were classified as moderate intensity work, and others were light physical activity. Edema was graded degree Ι if it was local, otherwise graded degree II. The residual GFR was measured at the third year of peritoneal dialysis initiation and calculated base on the formula recommended by Nolph [23]. The equation was: residual GFR = (renal urea clearance + renal creatinine clearance) /2, where renal urea clearance (ml/min) = (urine urea concentration/ serum urea concentration) ×24 h urine volume/1440, renal creatinine clearance (ml/min) = (urine creatinine concentration/ serum creatinine concentration) ×24 h urine volume/1 440.

Meanwhile, nutritional markers and biochemical parameters were measured including normalized protein catabolic rate (nPCR), albumin, total protein, hemoglobin, white blood cell count (WBC), red blood cell count (RBC), hematocrit, platelet, iron concentration (Fe^2+^), total iron binding capacity (TIBC), transferrin saturation (TSAT), ferritin, intact parathyroid hormone (iPTH), alkline phosphatase (AKP), serum concentration of Calcium (Ca^2+^), Kalium (K^+^), Phosphorus (P^3-^), Sodium (Na^+^), Chlorine (Cl^-^), Magnesium (Mg^2+^), blood glucose, triglyceride (TG), cholesterol (TC), low density lipoprotein (LDL), high density lipoprotein (HDL), aspertate aminotransferase (AST), alanine aminotransferase (ALT), carbon dioxide combining power (CO2-CP), urea, uric acid, creatinine and 24h urine volume. Protein catabolic rate (PCR) was calculated by the formula proposed by Tattersall [24] as follows: nPCR (mg/kg/day) = 1497×G/V +1.7, where G/V = [(Uv × Uc) + (Dv × Dc)]/1440V [Uv = volume of 24 h urine collection; Uc = urine urea concentration in 24 h urine collection; Dv = volume of 24 h collection of spent dialysate; Dc = urea concentration in 24 h collection of spent dialysate.

### Statistical analysis

Participants were classified into four groups based on quartiles of residual GFR (Q1, Q2, Q3, Q4), and comparisons among the four groups were conducted. Data were checked for normality first and presented as (mean ± standard deviation) or median (range) for continuous variables, while count (%) value for categorical variables. Comparisons were made by one-way ANOVA or Kruskal-Wallis H test for continuous variables while Cochran-Mantel-Haenszel χ^2^ test for categorical variables. The loss of residual renal function was defined as residual GFR less than 1 mL/min per 1.73m^2^. Logistic regression was used to estimate the odds ratio of RRF loss associated with serum biochemical parameters, setting the second quartile as a reference group. Furthermore, trends for the relationships between RRF and biochemical parameters were also tested by comparing the median of residual GFR among groups classified by the interquartile range of biochemical parameters.

The statistical analyses were performed using SAS software (version 9.2.3), and all P-values calculated as two-sided. The data was analyzed as normal distribution if the *P*- value was more than 0.10 while the association was considered significant if it was less than 0.05.

## Result

Basic characteristics of patients at study inclusion were given in Table 1. The mean age of the patients was (44.52±13.19) years old, in which 48.39% were males, 25.17% were high strength workers, and 35.48% have history of hypertension or diabetes. The median residual GFR was 1.68 mL/min per 1.73m^2^ (range, 0.00–12.32 mL/min per 1.73m^2^). The mean age of the four groups was not statistically different ((45.17±14.64) vs. (41.53±11.34) vs. (45.39±12.29) vs. (45.95±14.20), *P* = 0.450). And the distribution of residual GFR was not statistically different between male and female (*P* = 0.695), similar results were observed according to the profession of patients (*P* = 0.632), degree of education (*P* = 0.702) and the medical history of hypertension or diabetes (*P* = 0.091).

**Table 1:** Baseline characteristics of study subjects stratified by the interquartile range of residual GFR.

Variables	Total	residual GFR (mL/min per 1.73m^2^)^a^				F/χ^2^	*P* ^b^
		Q1 (0–0.36)	Q2 (0.36–1.68)	Q3 (1.68–2.99)	Q4 (2.99–12.32)		
**age**	44.52±13.19	45.17±14.64	41.53±11.34	45.39±12.29	45.95±14.20	0.89	0.450
**sex**						0.15	0.695
male	75(48.39%)	23(30.67%)	16(21.33%)	14(18.67%)	22(29.33%)		
female	80(51.61%)	16(20.00%)	22(27.50%)	25(31.25%)	17(21.25%)		
**occupation**						0.92	0.632
high strength work	36(25.17%)	9(25.00%)	10(27.78%)	12(33.33%)	5(13.89%)		
moderate intensity work	46(32.17%)	10(21.74%)	15(32.61%)	9(19.57%)	12(26.09%)		
light physical activity	61(42.66%)	18(29.51%)	9(14.75%)	14(22.95%)	20 (32.79%)		
**education**						0.71	0.702
primary school or below	37(24.50%)	12(32.43%)	7(18.92%)	10(27.03%)	8(21.62%)		
junior or high school	87(57.62%)	19(21.84%)	24(27.59%)	22(25.29%)	22(25.29%)		
college or above	27(17.88%)	7(25.93%)	6(22.22%)	5(18.52%)	9(33.33%)		
**hypertension** **or diabetes**						2.86	0.091
no	100(64.52%)	28(28.00%)	29(29.00%)	19(19.00%)	24(24.00%)		
yes	55(35.48%)	11(20.00%)	9(16.36%)	20(36.36%)	15(27.28%)		

^a^ classified into four groups based on quartiles of residual GFR.

^**b**^ Comparisons were made by one-way ANOVA or Kruskal-Wallis H test for continuous variables while Cochran-Mantel-Haenszel χ^2^test for categorical variables.

Table 2 showed the comparison of nutritional markers and biochemical parameters among the four groups classified by the interquartile range of residual GFR. Most nutritional markers and biochemical parameters were not statistically different among the four groups. However, the median of 24 hours urine volume in patients with higher residual GFR was significantly higher than patients with lower residual GFR (*P*<0.001). Similar results were found in nPCR (*P*<0.001). Inverse tendency was found in serum concentration of P^3-^ (*P* = 0.001) and creatinine (*P*<0.001). In addition, the mean values of BSA (*P* = 0.004) and the median of Na^+^ (*P*<0.001), Cl^-^ (*P*<0.001) and Mg^2+^ (*P*<0.001) were also found statistically different among the four groups.

**Table 2:** Comparisons of nutritional markers and biochemical parameters among the four groups stratified by the interquartile range of residual GFR.

**Variables**	**residual GFR (mL/min per 1.73m**^**2**^**)**^a^				**χ**^**2**^**/F/K**	***P***^b^
	**Q1 (0–0.36)**	**Q2 (0.36–1.68)**	**Q3 (1.68–2.99)**	**Q4 (2.99–12.32)**		
**BMI(kg/m**^**2**^**)**					1.48	0.477
<18.5	3(12.00%)	9(36.00%)	8(32.00%)	5(20.00%)		
18.5–23.9	31(28.44%)	27(24.77%)	23(21.10%)	28(25.69%)		
≥24	5(25.00%)	1(5.00%)	8(40.00%)	6(30.00%)		
**BSA(m**^**2**^**)**	1.65±0.16	1.53±0.13	1.57±0.17	1.64±0.17	4.61	0.004
**Edema**					5.69	0.058
No	31(29.25%)	28(26.42%)	25(23.58%)	22(20.75%)		
Ⅰ	5(14.71%)	6(17.65%)	11(32.35%)	12(35.29%)		
Ⅱ	3(21.43%)	4(28.57%)	3(21.43%)	4(28.57%)		
**nPCR (mg/kg.d)**	30.43(17.30–40.01)	31.54(18.18–54.28)	34.87(10.33–58.31)	36.32(22.62–83.09)	21.03	<0.001
**Albumin(g/L)**	37.70(29.90–45.50)	37.35(22.20–44.50)	37.90(16.90–47.60)	36.65(21.10–43.20)	0.67	0.881
**Total protein(g/L)**	68.80(57.40–78.90)	70.80(51.00–97.00)	68.20(46.80–83.40)	67.80(52.10–79.60)	2.08	0.556
**WBC(10**^**9**^**/L)**	5.95±1.68	6.36±1.88	6.62±2.05	6.18±1.58	0.94	0.425
**RBC(10**^**12**^**/L)**	3.25±0.71	3.22±0.55	3.41±0.57	3.39±0.57	1.00	0.394
**Hemoglobin(g/L)**	95.22±21.37	93.97±16.89	100.97±16.41	99.97±17.11	1.44	0.233
**Hematocrit(%)**	28.20±6.74	28.10±4.94	29.96±4.93	29.75±5.16	1.28	0.285
**Platelet(10**^**9**^**/L)**	176.08±67.19	204.38±54.20	200.64±69.52	191.97±56.80	1.56	0.202
**Fe**^**2+**^ **(μmol/L)**	10.22(1.90–35.04)	11.90(4.81–30.15)	12.11(1.73–23.63)	11.76(2.42–34.80)	1.78	0.620
**TIBC**	46.64±10.01	43.97±9.08	41.98±8.70	41.26±8.53	2.62	0.053
**TSAT**	20.72(2.35–75.90)	26.08(11.08–85.41)	29.27(5.16–69.24)	30.73(8.96–77.51)	6.61	0.085
**Ferritin(μg/L)**	158.00(10.10–696.00)	126.90(13.90–766.10)	143.75(18.00–980.00)	187.80(35.80–979.80)	1.80	0.614
**iPTH (ng/L)**	398.70(38.72–1106.00)	311.35(85.51–1182.00)	315.20(29.40–1198.00)	313.70(19.14–1281.00)	1.97	0.578
**AKP (U/L)**	77.00(41.00–183.00)	74.00(34.00–385.00)	80.00(40.00–167.00)	74.00(33.00–239.00)	1.51	0.681
**Ca**^**2+**^**(mmol/L)**	2.36(1.78–2.66)	2.36(1.58–3.02)	2.29(1.64–2.74)	2.27(2.05–3.05)	3.72	0.294
**P**^**3-**^**(mmol/L)**	2.03(1.01–3.40)	1.66(0.56–3.22)	1.54(0.80–2.73)	1.52(0.46–2.57)	17.14	0.001
**K**^**+**^**(mmol/L)**	4.07(2.61–5.79)	4.22(3.33–5.48)	4.02(2.68–5.98)	4.22(2.69–5.84)	0.95	0.814
**Na**^**+**^**(mmol/L)**	139.00(131.10–148.40)	138.25(132.90–142.70)	138.70(125.60–142.70)	140.80(131.80–144.80)	22.87	<0.001
**Cl**^**-**^**(mmol/L)**	95.60(87.00–135.80)	94.30(85.30–105.00)	96.50(81.90–105.80)	99.60(88.80–105.00)	33.97	<0.001
**Mg**^**2+**^**(mmol/L)**	1.01(0.69–1.29)	0.84(0.63–1.26)	0.82(0.59–1.31)	0.89(0.62–1.31)	19.46	<0.001
**Blood glucose(mmol/L)**	5.60(4.24–28.49)	5.24(4.05–8.82)	5.40(3.79–10.59)	5.40(4.51–11.62)	5.06	0.167
**TG(mmol/L)**	1.25(0.26–5.95)	1.08(0.34–4.15)	1.30(0.23–7.77)	1.29(0.50–5.97)	3.03	0.387
**TC(mmol/L)**	4.49 (2.58–6.75)	4.59(2.84–8.26)	4.61(2.87–8.32)	4.62(1.88–6.74)	1.72	0.633
**LDL(mmol/L)**	2.64(1.05–4.59)	2.46(1.11–5.22)	2.62(1.06–5.44)	2.29(0.50–4.23)	3.47	0.325
**HDL(mmol/L)**	1.06(0.68–10.50)	1.13(0.62–1.95)	1.06(0.51–1.80)	1.07(0.62–2.33)	1.04	0.791
**AST(U/L)**	15.00(7.00–42.00)	16.00(8.00–28.00)	18.00(6.00–53.00)	15.00(8.00–26.00)	4.36	0.225
**ALT(U/L)**	14.00(6.00–66.00)	12.00(4.00–29.00)	12.00(1.00–85.00)	13.00(6.00–42.00)	1.48	0.687
**Total bilirubin(μmol/L)**	4.80(1.10–11.00)	5.25(1.00–10.80)	4.60(0.80–12.00)	4.10(0.60–15.20)	1.69	0.640
**CO2-CP(mmol/L)**	25.70(19.20–31.70)	26.45(5.20–33.60)	25.40(10.60–31.30)	25.60(6.50–35.60)	1.75	0.627
**Urea(mmol/L)**	20.10(8.78–34.76)	18.55(8.77–28.24)	15.83(8.17–37.52)	16.34(5.61–28.65)	6.88	0.076
**Uric acid (μmol/L)**	406.57. ±86.47	381.90±83.70	406.95±91.85	433.19±80.11	2.30	0.079
**Creatinine (μmol/L)**	1184.00(714.00–1724.00)	1013.00(633.00–1736.00)	826.00(398.00–1738.00)	813.00(423.00–1623.00)	31.64	<0.001
**24h urine volume(mL)**	0.03(0.00–400.00)	400.00(150.00–1100.00)	860.00(350.00–1700.00)	1100.00(50.00–2400.00)	110.71	<0.001

^a^ classified into four groups based on quartiles of residual GFR.

^**b**^ Comparisons were made by one-way ANOVA or Kruskal-Wallis H test for continuous variables while Cochran-Mantel-Haenszel χ^2^test for categorical variables.

Given to potential confounding effects, age and sex were adjusted in the logistic regression model for each of the variables above with *P* value less than 0.05. It showed that higher was always better for nPCR, serum concentration of Cl^-^ and 24 hours urine volume. Compared to Q2, patients with nPCR among Q3 had a lower risk of RRF loss (OR = 0.24, 95%CI: 0.09–0.65), and 0.11 (95%CI: 0.03–0.35) for patients among Q4. The low level of Cl^-^ was a risk factor for RRF loss (OR_Q1_ = 5.34, 95%CI: 1.94–14.68) while a protective factor (OR = 0.28, 95%CI: 0.09–0.85) when it was among Q4. For 24 hours urine volume, the risk of RRF loss would increase to 22.87 times (95%CI: 2.76–189.24) when it was among Q1, while decreased to 0.08 (95%CI: 0.02–0.28) when it was more than 550mL compared to the second quartile (200-550mL). For serum P^3-^, Mg^2+^ and creatinine, higher was always worse. Compared to the second quartile, patients with P^3-^ concentration among Q3 had higher risk of RRF deterioration (OR = 2.67, 95%CI: 1.03–6.92) and 3.45 (95%CI: 1.35–9.04) for patients among Q4. And the risk increased to 3.77(95%CI: 1.48–3.63) when serum Mg^2+^ was among Q3. Similar results were found in serum creatinine (OR_Q3_ = 2.93, 95%CI: 1.09–7.88; OR_Q4_ = 8.64, 95%CI: 2.79–26.78). There was no relationship between RRF deterioration and BSA and serum Na^+^. The result was showed in Table 3.

**Table 3:** The odds ratio of RRF loss associated with nutritional markers and biochemical parameters.

**Variables**	**OR(95%CI)**^a^	**Ward χ2**	***P***
**BSA(m**^**2**^**)**			
Q1 (1.21–1.47)	0.55(0.20–1.51)	1.33	0.248
Q2 (1.47–1.60)	1		
Q3 (1.60–1.73)	1.14(0.44–2.99)	0.07	0.785
Q4 (1.73–2.02)	0.57(0.20–1.64)	1.09	0.296
**nPCR(mg/kg.d)**			
Q1 (10.33–28.79)	1.06(0.42–2.70)	0.02	0.899
Q2 (28.79–33.24)	1		
Q3 (33.24–38.98)	0.24(0.09–0.65)	7.99	0.005
Q4 (38.98–83.09)	0.11(0.03–0.35)	13.76	<0.001
**P**^**3-**^**(mmol/L)**			
Q1 (0.46–1.30)	0.69(0.24–2.01)	0.46	0.499
Q2 (1.30–1.66)	1		
Q3 (1.66–2.11)	2.67(1.03–6.92)	4.07	0.044
Q4 (2.11–3.40)	3.45(1.35–9.04)	6.61	0.010
**Na**^**+**^**(mmol/L)**			
Q1 (125.60–137.40)	2.32(0.92–5.89)	3.16	0.076
Q2 (137.40–139.40)	1		
Q3 (139.40–141.00)	0.98(0.38–2.51)	0.01	0.960
Q4 (141.00–148.40)	0.55(0.21–1.44)	1.46	0.227
**Cl**^**-**^**(mmol/L)**			
Q1 (81.90–93.90)	5.34(1.94–14.68)	10.55	0.001
Q2 (93.90–96.40)	1		
Q3 (96.40–99.30)	0.82(0.32–2.08)	0.18	0.671
Q4 (99.30–135.80)	0.28(0.09–0.85)	5.03	0.025
**Mg**^**2+**^**(mmol/L)**			
Q1 (0.62–0.79)	1.55(0.58–4.16)	0.77	0.381
Q2 (0.79–0.89)	1		
Q3 (0.89–1.01)	2.49(0.94–6.56)	3.39	0.066
Q4 (1.01–1.31)	3.77(1.48–3.63)	7.70	0.006
**Creatinine(μmol/L)**			
Q1 (398.00–795.00)	0.35(0.10–1.18)	2.87	0.090
Q2 (795.00–963.00)	1		
Q3 (963.00–1214.00)	2.93(1.09–7.88)	4.56	0.033
Q4 (1214.00–1738.00)	8.64(2.79–26.78)	13.95	<0.001
**24h urine volume(mL)**			
Q1 (0.00–200.00)	22.87(2.76–189.24)	8.42	0.004
Q2 (200.00–550.00)	1		
Q3 (550.00–1000.00)	0.08(0.02–0.28)	15.82	<0.001
Q4 (1000.00–2400.00)	--	0.01	0.940

^a^ age and sex were adjusted in the logistic regression model for each of the variables.

Table 4 showed that residual GFR levels increased with nPCR rising. The medians of residual GFR were 0.70, 0.57, 2.37, and 2.24 mL/min per 1.73m^2^ in nPCR quartiles (*P* for trend <0.0001). And similar results were noted in serum Cl^-^ (*P* <0.0001), and 24 hours urine volume (*P* <0.0001). There was a drop tendency of residual GFR with the increase of serum P^3-^ (*P* for trend less than 0.001), Mg^2+^ (*P* = 0.025) and creatinine (*P* <0.0001), respectively.

**Table 4:** The trend of the relationship between residual renal function and biochemical parameters.

Variables	residual GFR (median)^a^				*P*
	Q1(0–25%)	Q2(25%-50%)	Q3(50%-75%)	Q4(75%-100%)	
**nPCR**	0.70	0.57	2.37	2.24	<0.001
**P**^**3-**^	2.46	1.97	0.91	0.57	<0.001
**Cl**^**-**^	0.48	1.39	1.70	3.66	<0.001
**Mg**^**2+**^	2.06	1.92	1.23	0.49	0.025
**creatinine**	2.78	1.74	1.07	0.48	<0.001
**24h urine volume**	0.00	0.77	2.30	3.35	<0.001

^a^ the median of residual GFR among groups classified by the interquartile range of biochemical parameters.

## Discussion

As an important prognostic factor for PD patients, higher RRF is better for disease progression. Preserving RRF therefore became a key target in the treatment of CAPD patients. The present study showed a significant correlation between the fall in RRF and the increase in serum P^3-^(OR_Q3_ = 2.67, 95%CI: 1.03–6.92; OR_Q4_ = 3.45, 95%CI: 1.35–9.04), Mg^2+^ (OR_Q4_ = 3.77, 95%CI: 1.48–3.63), and creatinine (OR_Q3_ = 2.93, 95%CI: 1.09–7.88; OR_Q4_ = 8.64 95%CI: 2.79–26.78), while positively associated with nPCR (OR_Q3_ = 0.24, 95%CI: 0.09–0.65; OR_Q4_ = 0.11, 95%CI: 0.03–0.35), 24 hours urine volume(OR_Q1_ = 22.87, 95%CI: 2.76–189.24; OR_Q3_ = 0.08, 95%CI: 0.02–0.28) and serum Cl^-^ (OR_Q1_ = 5.34, 95%CI: 1.94–14.68; OR_Q4_ = 0.28, 95%CI: 0.09–0.85), respectively.

The positive relationship between RRF and 24h urine volume may due to the fact that residual renal contributed to urinary formation and micturition excretory. And the higher risk of RRF deterioration induced by increased serum creatinine and Mg^2+^ may result from the fact that loss of RRF would contributed to excretory impairment of body fluid and eleclxolytes.

Abnormal calcium-phosphate metabolism such as hyperphosphatemia is a frequent complication in PD patients as it is in HD patients. According to the NECOSAD study, around 40% of the long-term PD patients had serum phosphorus level above the Kidney Disease Outcome Quality Initiative (K/DOQI) target (1.78 mmol/L) [25]. RRF is one of the key determinants of phosphate control in PD patients and its importance outweighs that of the PD clearance among those with preserved RRF [26, 27]. It has been reported that a significantly lower phosphate correlated with a higher RRF, and the RRF in PD patients contributes significantly to the maintenance of phosphate balance and may explain the lower prevalence of cardiac valve calcification (CVC) in PD patients [28]. Cardiac valve calcification has long been regarded as a consequence of abnormal calcium-phosphate metabolism, which was an important complication in dialysis patients and was largely attributed to abnormally increased calcium and phosphorus product. The poor phosphorus control together with the greater inflammatory response in anuric peritoneal dialysis patients translated to a greater calcification risk profile and thus predisposed to a higher incidence of valvular calcification [27].

The relationship between RRF and serum chlorine level should be evaluated with caution. In consideration of the fact that patients with ESRD tended to be educated to limit salt intake in our hospital, we can't rule out the possibility that patients with little residual GFR restricted chlorine salt intake more severely. In addition, residual GFR loss would lead to more serious fluid overload, which need to remove much more water and molecules by dialysis, including chlorine, to maintain fluid homeostasis than patients with relative high renal GFR.

Normalized protein catabolic rate (nPCR) is considered a nutritional marker for nitrogen intake and a useful measure to evaluate dietary protein intake in patients with ESRD. Its increase can be obtained by means of intra-dialytic parenteral nutrition [29–31]. It has been reported that nPCR correlated well with RRF, a significant reduction of nPCR occurs in progressive renal perfusion insufficiency, and may predict the need for dialysis treatment. It has been reported that the level of nPCR less than 0.8 at initiation predicted future lower nPCR levels and mortality on dialysis [32, 33]. The underlying mechanisms for positive relationship between nPCR levels and residual GFR were that low nPCR levels predicted weight loss and protein calorie malnutrition, which were predictors of morbidity and mortality for patients with chronic renal failure and CAPD [34–36]. Another genuine physiological association was that reduced renal function leaded to insufficient protein intake and decreased nPCR level. In addition, it has been reported that nPCR was positively correlated with normalized models of dialysis adequacy including KT/V (urea), total weekly creatinine clearance and the dialysis index [33, 37].

The present study also has some limitations. First, the number of patients was relatively small. Second, it was an exploratory study of the association between nutritional markers and biochemical parameters and residual GFR. Finally, although the univariate logistic regression analysis performed in this study indicated that serum P^3-^, Ca^2+^, Cl^-^, Mg^2+^, creatinine, nPCR levels and the 24 hours urine volume as independent factors for the residual GFR, which could not exclude the impact of interactions among these variables. Accordingly, the results of this study should be confirmed by large-scale prospective and more rigorous studies.

Overall, the present study demonstrated that serum P^3-^, Cl^-^, Mg^2+^, creatinine and the 24 hours urine volume were significantly associated with residual GFR. As an important nutritional marker, the nPCR level may serve as a valuable preditor of residual renal function loss.

## Supporting Information

S1 Appendix STROBE checklist—checklist of items that should be included in reports of observational studies. (DOCX)

S2 Appendix The data set of demographic characteristics, nutritional markers and biochemical parameters of the 155 participants. (XLSX)

## References

[1] Coresh J, Selvin E, Stevens LA, Manzi J, Kusek JW, Eggers P, et al Prevalence of chronic kidney disease in the United States. JAMA. 2007;298(17):2038–2047. 17986697 10.1001/jama.298.17.2038

[2] Stengel B, Combe C, Jacquelinet C, Briancon S, Fouque D, Laville M, et al The French Chronic Kidney Disease-Renal Epidemiology and Information Network (CKD-REIN) cohort study. Nephrol Dial Transplant. 2014;29(8):1500–1507. 10.1093/ndt/gft388 24064325 PMC4106639

[3] Hallan SI, Coresh J, Astor BC, Asberg A, Powe NR, Romundstad S, et al International comparison of the relationship of chronic kidney disease prevalence and ESRD risk. J Am Soc Nephrol. 2006;17(8):2275–2284. 16790511 10.1681/ASN.2005121273

[4] Zhang L, Wang F, Wang L, Wang W, Liu B, Liu J, et al Prevalence of chronic kidney disease in China: a cross-sectional survey. Lancet. 2012;379(9818):815–822. 10.1016/S0140-6736(12)60033-6 22386035

[5] Diaz-Buxo JA, White SA, Himmele R. The importance of residual renal function in peritoneal dialysis patients. Adv Perit Dial. 2013;29:19–24. 24344485

[6] Maiorca R, Brunori G, Zubani R, Cancarini GC, Manili L, Camerini C, et al Predictive value of dialysis adequacy and nutritional indices for mortality and morbidity in CAPD and HD patients. A longitudinal study. Nephrol Dial Transplant. 1995;10(12):2295–2305. 8808229 10.1093/ndt/10.12.2295

[7] Rocco M, Soucie JM, Pastan S, McClellan WM. Peritoneal dialysis adequacy and risk of death. Kidney Int. 2000;58(1):446–457. 10886593 10.1046/j.1523-1755.2000.00184.x

[8] Bargman JM, Thorpe KE, Churchill DN. Relative contribution of residual renal function and peritoneal clearance to adequacy of dialysis: a reanalysis of the CANUSA study. J Am Soc Nephrol. 2001;12(10):2158–2162. 11562415 10.1681/ASN.V12102158

[9] Szeto CC, Wong TY, Leung CB, Wang AY, Law MC, Lui SF, et al Importance of dialysis adequacy in mortality and morbidity of chinese CAPD patients. Kidney Int. 2000;58(1):400–407. 10886588 10.1046/j.1523-1755.2000.00179.x

[10] Amici G, Virga G, Da Rin G, Grandesso S, Vianello A, Gatti P, et al Serum beta-2-microglobulin level and residual renal function in peritoneal dialysis. Nephron. 1993;65(3):469–471. 8290003 10.1159/000187533

[11] McCarthy JT, Williams AW, Johnson WJ. Serum beta 2-microglobulin concentration in dialysis patients: importance of intrinsic renal function. J Lab Clin Med. 1994;123(4):495–505. 8144998

[12] Ates K, Nergizoglu G, Keven K, Sen A, Kutlay S, Erturk S, et al Effect of fluid and sodium removal on mortality in peritoneal dialysis patients. Kidney Int. 2001;60(2):767–776. 11473661 10.1046/j.1523-1755.2001.060002767.x

[13] Konings CJ, Kooman JP, Schonck M, Dammers R, Cheriex E, Palmans Meulemans AP, et al Fluid status, blood pressure, and cardiovascular abnormalities in patients on peritoneal dialysis. Perit Dial Int. 2002;22(4):477–487. 12322819

[14] Konings CJ, Kooman JP, Schonck M, Struijk DG, Gladziwa U, Hoorntje SJ, et al Fluid status in CAPD patients is related to peritoneal transport and residual renal function: evidence from a longitudinal study. Nephrol Dial Transplant. 2003;18(4):797–803. 12637651 10.1093/ndt/gfg147

[15] Park HC, Lee H, Lee JP, Kim DK, Oh KH, Joo KW, et al Lower residual renal function is a risk factor for depression and impaired health-related quality of life in Korean peritoneal dialysis patients. J Korean Med Sci. 2012;27(1):64–71. 10.3346/jkms.2012.27.1.64 22219616 PMC3247777

[16] Pecoits-Filho R, Heimburger O, Barany P, Suliman M, Fehrman-Ekholm I, Lindholm B, et al Associations between circulating inflammatory markers and residual renal function in CRF patients. Am J Kidney Dis. 2003;41(6):1212–1218. 12776273 10.1016/s0272-6386(03)00353-6

[17] van de Kerkhof J, Schalkwijk CG, Konings CJ, Cheriex EC, van der Sande FM, Scheffer PG, et al Nepsilon-(carboxymethyl)lysine, Nepsilon-(carboxyethyl)lysine and vascular cell adhesion molecule-1 (VCAM-1) in relation to peritoneal glucose prescription and residual renal function; a study in peritoneal dialysis patients. Nephrol Dial Transplant. 2004;19(4):910–916. 15031349 10.1093/ndt/gfh004

[18] Yamada S, Tsuruya K, Taniguchi M, Yoshida H, Tokumoto M, Hasegawa S, et al Relationship between residual renal function and serum fibroblast growth factor 23 in patients on peritoneal dialysis. Ther Apher Dial. 2014;18(5):383–390. 10.1111/1744-9987.12170 24674095

[19] Weisbord SD, Bernardini J, Mor MK, Hartwig KC, Nicoletta PJ, Palevsky PM, et al The effect of coronary angiography on residual renal function in patients on peritoneal dialysis. Clin Cardiol. 2006;29(11):494–497. 17133846 10.1002/clc.4960291105 PMC6654194

[20] Chang TI, Kang EW, Kim HW, Ryu GW, Park CH, Park JT, et al Low Serum Bicarbonate Predicts Residual Renal Function Loss in Peritoneal Dialysis Patients. Medicine. 2015;94(31):e1276 10.1097/MD.0000000000001276 26252296 PMC4616581

[21] Maeda Y, Yoshida S, Hirai T, Kawasaki T, Kuyama T. Estimated glomerular filtration rate -a more stable indicator than creatinine clearance in peritoneal dialysis practice. J Rural Med. 2013;8(1):171–175. 10.2185/jrm.8.171 25649632 PMC4309341

[22] George SL, Gehan EA. Methods for measurement of body surface area. J Pediatr. 1979;94(2):342–343. 762643 10.1016/s0022-3476(79)80870-7

[23] Nolph KD, Moore HL, Prowant B, Meyer M, Twardowski ZJ, Khanna R, et al Cross sectional assessment of weekly urea and creatinine clearances and indices of nutrition in continuous ambulatory peritoneal dialysis patients. Perit Dial Int. 1993;13:178–183. 8369345

[24] Tattersall J, Greenwood RN, Farrington K. Adequacy of dialysis In: Davison AM, Cameron JS, Grunfeld J-P, Kerr DNS, Ritz E, Winnearls CG, eds. Oxford Textbook of Nephrology. Oxford University Press: 1998; 2074–2087.

[25] Noordzij M, Korevaar JC, Bos WJ, Boeschoten EW, Dekker FW, Bossuyt PM, et al Mineral metabolism and cardiovascular morbidity and mortality risk: peritoneal dialysis patients compared with haemodialysis patients. Nephrol Dial Transplant. 2006;21(9):2513–2520. 16799173 10.1093/ndt/gfl257

[26] Wang AY, Woo J, Sea MM, Law MC, Lui SF, Li PK. Hyperphosphatemia in Chinese peritoneal dialysis patients with and without residual kidney function: what are the implications? Am J Kidney Dis. 2004;43(4):712–720. 15042549

[27] Wang AY, Lai KN. The importance of residual renal function in dialysis patients. Kidney Int. 2006;69(10):1726–1732. 16612329 10.1038/sj.ki.5000382

[28] Rroji M, Seferi S, Cafka M, Petrela E, Likaj E, Barbullushi M, et al Is residual renal function and better phosphate control in peritoneal dialysis an answer for the lower prevalence of valve calcification compared to hemodialysis patients? Int Urol Nephrol. 2014;46(1):175–1782. 10.1007/s11255-013-0438-7 23591721

[29] Aparicio M, Cano N, Chauveau P, Azar R, Canaud B, Flory A, et al Nutritional status of haemodialysis patients: a French national cooperative study. French Study Group for Nutrition in Dialysis. Nephrol Dial Transplant. 1999;14(7):1679–1686. 10435876 10.1093/ndt/14.7.1679

[30] Chauveau P, Naret C, Puget J, Zins B, Poignet JL. Adequacy of haemodialysis and nutrition in maintenance haemodialysis patients: clinical evaluation of a new on-line urea monitor. Nephrol Dial Transplant. 1996;11(8):1568–1573. 8856213

[31] Pollock CA, Ibels LS, Zhu FY, Warnant M, Caterson RJ, Waugh DA, et al Protein intake in renal disease. J Am Soc Nephrol. 1997;8(5):777–783. 9176847 10.1681/ASN.V85777

[32] Kopple JD, Greene T, Chumlea WC, Hollinger D, Maroni BJ, Merrill D, et al Relationship between nutritional status and the glomerular filtration rate: results from the MDRD study. Kidney Int. 2000;57(4):1688–1703. 10760105 10.1046/j.1523-1755.2000.00014.x

[33] Chandna SM, Kulinskaya E, Farrington K. A dramatic reduction of normalized protein catabolic rate occurs late in the course of progressive renal insufficiency. Nephrol Dial Transplant. 2005;20(10):2130–2138. 15956057 10.1093/ndt/gfh940

[34] Ikizler TA, Greene JH, Wingard RL, Parker RA, Hakim RM. Spontaneous dietary protein intake during progression of chronic renal failure. J Am Soc Nephrol. 1995;6(5):1386–1391. 8589313 10.1681/ASN.V651386

[35] Ikizler TA, Wingard RL, Harvell J, Shyr Y, Hakim RM. Association of morbidity with markers of nutrition and inflammation in chronic hemodialysis patients: a prospective study. Kidney Int. 1999;55(5):1945–1951. 10231458 10.1046/j.1523-1755.1999.00410.x

[36] Adequacy of dialysis and nutrition in continuous peritoneal dialysis: association with clinical outcomes. Canada-USA (CANUSA) Peritoneal Dialysis Study Group. J Am Soc Nephrol. 1996;7(2):198–207. 8785388 10.1681/ASN.V72198

[37] Lutes R, Holley JL, Perlmutter J, Piraino B. Correlation of normalized protein catabolic rate to weekly creatinine clearance and KT/V in patients on peritoneal dialysis. Adv Perit Dial. 1993;9:97–100. 8105973

